# Mucoid Degeneration of the Anterior Cruciate Ligament Treated with Selective Arthroscopic Debridement: A Case Report with 54-Month Follow-Up

**DOI:** 10.3390/reports9040324

**Published:** 2026-09-25

**Authors:** Hassan Zmerly, Federica Dellafiore, Silvio Caravelli, Massimiliano Mosca, Luigi Di Lorenzo

**Affiliations:** 1 Department of Life Sciences, Health, and Health Professions, Link Campus University, 00165 Rome, Italy; 2 Department of Orthopedics, Villa Erbosa Hospital, GSD, 40129 Bologna, Italy; 3 Department of Orthopaedics Bentovoglio, IRCCS Istituto Ortopedico Rizzoli, 40136 Bologna, Italy

**Keywords:** anterior cruciate ligament, mucoid degeneration, arthroscopy, debridement, knee pain

## Abstract

**Background and Clinical Significance:** Anterior cruciate ligament (ACL) mucoid degeneration is a rare, non-traumatic condition characterized by the deposition of mucoid material within the ligament, resulting in hypertrophy and potential impingement against surrounding intra-articular structures; **Case Presentation:** A 43-year-old man presented with knee pain, swelling, and restricted range of motion. Magnetic resonance imaging demonstrated mucoid degeneration of the ACL associated with mild articular degenerative changes, without evidence of a meniscal tear. Following the failure of conservative management, the patient underwent knee arthroscopy. Intraoperative findings revealed an enlarged ACL with impingement against the intercondylar notch and the lateral femoral condyle. The patient was treated with arthroscopic ACL debridement, which resulted in adequate decompression. At the 54-month follow-up, the patient remained pain-free, had regained full range of motion, and showed no clinical instability. The Lysholm score improved from 41 preoperatively to 95 postoperatively; **Conclusions:** Mucoid degeneration of the ACL is a rare but important cause of knee pain and restricted range of motion. When conservative treatment fails, arthroscopic management can provide symptom resolution and restore range of motion. However, careful preservation of healthy ACL fibers during debridement is a key technical consideration for achieving symptom relief while maintaining ligament function and knee stability. This case suggests that selective arthroscopic debridement may provide sustained symptom relief while preserving ACL stability in patients with isolated symptomatic mucoid degeneration, with favorable outcomes maintained at long-term follow-up.

## 1. Introduction and Clinical Significance

Anterior cruciate ligament mucoid degeneration (ACL-MD) is an uncommon disease, primarily affecting individuals in their fourth decade of life. It is characterized by the deposition of mucoid substance within the collagen bundles of the ligament, leading to hypertrophy [1,2,3]. This condition may cause knee pain and restricted range of motion due to impingement between the enlarged ACL and surrounding structures, typically without knee instability. In many cases, these lesions are associated with concomitant intra-articular injuries that should be addressed during treatment [4,5].

The initial approach consists of conservative treatment aimed at relieving pain and restoring joint mobility, including pharmacological therapy, intra-articular injections, osteopathy, physical therapy and rehabilitation [6]. When conservative management fails to provide adequate symptom relief, arthroscopic treatment is commonly considered for patients with persistent symptoms [7].

We report a rare case of symptomatic ACL mucoid degeneration with extensive intra-ligamentous mucoid accumulation in the absence of other intra-articular pathologies that could account for the patient’s symptoms. After the failure of conservative treatment, the patient was treated with arthroscopic ACL debridement, achieving a reduction in the ACL volume without residual impingement or laxity. This case documents a durable medium- to long-term favorable outcome following selective debridement without the need for complete ACL resection or reconstruction.

## 2. Case Presentation

### 2.1. History

A previously healthy 43-year-old man with no relevant previous medical history presented to our orthopedic clinic with progressive, persistent left knee pain that had begun 12 months earlier. He reported anterior knee pain associated with swelling and restricted knee motion, with no history of trauma. The patient was 176 cm tall and weighed 85 kg, corresponding to a BMI of 27.4 kg/m^2^. He worked as a construction worker in a physically demanding occupation. Initially, his symptoms were predominantly mechanical and activity-related, particularly following physical exertion. Over time, the symptoms progressed and also occurred at rest, with recurrent knee swelling and increasing limitation of motion.

Over approximately 12 months, the patient underwent conservative management, including nonsteroidal anti-inflammatory drugs (NSAIDs), intra-articular hyaluronic acid injections, a course of oral corticosteroids, osteopathic treatment, physical therapy, and rehabilitation. These treatments provided only temporary symptom relief, with subsequent recurrence of pain, swelling, and limitation of knee motion.

Physical examination revealed knee pain and mild swelling, with a 5° extension deficit and flexion limited to 90°. Meniscal signs were positive, whereas the Lachman, varus and valgus stress tests were negative. The pivot-shift test could not be performed because of pain and knee motion limitation.

### 2.2. Investigation

Plain radiographs of the left knee in a standing position showed normal bone morphology and preserved joint spaces, with no malalignment or detectable abnormalities.

Magnetic resonance imaging (MRI) demonstrated mucoid degeneration of the anterior cruciate ligament (ACL). Sagittal, coronal, and axial MRI sequences revealed diffuse thickening and hypertrophy of the ACL with increased intra-ligamentous signal intensity while preserving the continuity and orientation of the ligament fibers, findings consistent with mucoid degeneration (Figure 1). The enlarged ACL occupied the intercondylar notch and contributed to mechanical impingement within it. Associated joint effusion and mild degenerative articular changes were also observed. No meniscal tears, hypertrophic synovial plicae, or intra-articular masses were present. No evidence of complete ACL rupture was identified.

Laboratory investigations for systemic inflammatory and rheumatic disorders were within normal limits.

### 2.3. Surgical Treatment

Following the failure of conservative treatment, the patient underwent arthroscopic surgery under spinal anesthesia after a thorough discussion of the procedure and provided written informed consent for surgery. Intraoperatively, marked hypertrophy with an area of mucoid degeneration of the ACL was identified, with intra-ligamentous mucoid changes clearly visible (Figure 2A). Full knee extension was restricted due to impingement of the hypertrophied ACL within the intercondylar notch. Both menisci were intact, with no evidence of pathological synovial plicae. Arthroscopic examination also revealed fibrillary chondromalacia involving the patellar and trochlear articular surfaces.

Arthroscopic debridement and decompression of the hypertrophic ACL were performed using the 90-degree knee position and the figure-of-four position. This resulted in adequate decompression (Figure 2B), with restoration of complete range of motion and sufficient space within the intercondylar notch. Mild cartilage shaving was performed. During arthroscopic debridement, the ACL was repeatedly assessed by probing to ensure preservation of its integrity. At the end of the procedure, intraoperative Lachman and pivot-shift tests demonstrated no evidence of laxity.

No histological examination was performed.

### 2.4. Follow-Up

No postoperative complications were observed. The patient immediately began a rehabilitation program consisting of range-of-motion exercises, with immediate restoration of full extension and progressive recovery of flexion, hamstring stretching, and quadriceps strengthening exercises. Immediate weight-bearing was allowed as tolerated, initially with the use of two crutches, which were gradually discontinued.

At the three-, 12-, and 54-month follow-up evaluations, the patient remained in good clinical condition. Physical examination revealed no knee swelling, and the patient reported complete resolution of the preoperative symptoms. Range of motion was fully restored, with full extension and flexion comparable to the contralateral side.

Functional outcomes improved markedly, with no evidence of postoperative instability, as demonstrated by negative Lachman and pivot-shift tests at follow-up. The Lysholm knee score increased from 41 points preoperatively to 95 points postoperatively. The patient returned to normal daily activities without limitations and expressed high satisfaction with the surgical outcome.

## 3. Discussion

Mucoid degeneration of the anterior cruciate ligament (ACL) is an uncommon, atraumatic, and often underrecognized condition characterized by mucoid deposition within the ligament, resulting in hypertrophy and potential impingement against surrounding intra-articular structures [8,9].

Patients typically present with knee pain, swelling, and progressive restriction of range of motion, particularly the loss of full knee flexion [10]. The diagnosis can be challenging because the clinical presentation is often nonspecific and may mimic other intra-articular pathologies. The differential diagnosis included other intra-articular lesions that may cause mechanical symptoms, such as meniscal tears, synovial plicae, loose bodies, osteochondral lesions, and intra-articular masses [10,11].

As with other ACL pathologies, magnetic resonance imaging plays a central role in identifying the characteristic features of ACL mucoid degeneration, including ligament enlargement, increased intra-ligamentous signal intensity—the so-called “celery stalk” sign—and preservation of fiber continuity. However, ACL mucoid degeneration may represent an incidental MRI finding when other intra-articular abnormalities are responsible for the patient’s knee symptoms.

In patients presenting with knee pain and restricted range of motion, initial management is generally conservative and may include physical therapy, osteopathy, anti-inflammatory medications, and intra-articular injections [6]. Surgical intervention is reserved for patients who remain symptomatic despite appropriate conservative treatment [12,13].

Arthroscopic treatment is considered the gold standard for refractory cases [14,15,16,17]. The first step is a comprehensive diagnostic arthroscopy to identify any associated intra-articular lesions that may account for the patient’s knee symptoms and to assess ACL stability and the extent of mucoid degeneration. Surgical management should be tailored according to the arthroscopic findings.

In the present case, conservative treatment failed to provide adequate symptom relief, and the patient continued to experience persistent pain and significant limitation of knee motion. Arthroscopic evaluation revealed marked ACL hypertrophy, with impingement against the intercondylar notch and lateral femoral condyle, as well as extensive mucoid degeneration. Other intra-articular pathologies that could account for the mechanical symptoms or limited range of motion, such as meniscal lesions, pathological synovial plicae, osteochondral defects, loose bodies, and intra-articular masses, were excluded. Only fibrillary chondromalacia involving the patellar and trochlear articular surfaces were observed and treated with limited shaving. ACL debridement was performed to remove the degenerated mucoid tissue, reduce ligament volume, and eliminate impingement between the hypertrophic ACL and the surrounding structures, without the need for notchplasty.

When performing selective ACL debridement, particular care should be taken to preserve as many intact ACL fibers as possible to maintain ligament function and postoperative knee stability. Excessive resection may increase the risk of postoperative instability and negatively affect functional outcomes. The extent of ACL resection remains an important consideration in the arthroscopic treatment of symptomatic mucoid degeneration [11]. Partial or selective debridement aims to remove the degenerative mucoid tissue and reduce ligament volume sufficiently to eliminate impingement while preserving as many functional ACL fibers as possible. This approach has the potential advantage of maintaining native ligament continuity, proprioceptive function, and knee stability, thereby avoiding the morbidity and additional rehabilitation associated with ACL reconstruction [18,19]. However, insufficient debridement may result in persistent impingement or incomplete symptom relief and may theoretically increase the risk of symptom recurrence [9].

In our case, selective arthroscopic debridement was performed while preserving as much healthy ACL tissue as possible. At the end of the procedure, the residual ligament demonstrated satisfactory continuity and stability.

Orthobiologic treatments have been recently proposed as adjunctive therapies following arthroscopic debridement in patients with concomitant degenerative joint changes [20]. In a clinical case study, Sesto et al. reported a satisfactory outcome following debridement and PRP injection [21]; however, further studies are needed to establish its clinical efficacy and define its role in the management of this condition.

The present case confirms the findings reported in the literature, demonstrating excellent clinical and functional outcomes following selective arthroscopic ACL debridement. However, the available evidence remains limited to case reports and small case series with short-term follow-up. At the 54-month follow-up, our patient remained pain-free, had regained full knee range of motion, and showed no evidence of postoperative instability, with negative Lachman and pivot-shift tests. Functional outcomes improved markedly, with the Lysholm knee score increasing from 41 preoperatively to 95 postoperatively. Given the favorable clinical condition, further follow-up imaging (e.g., MRI) was not performed, as it was not considered clinically necessary. These findings support the long-term effectiveness and safety of arthroscopic decompression while preserving the native ACL and maintaining knee stability.

## 4. Conclusions

Mucoid degeneration of the ACL should be considered in patients presenting with atraumatic knee pain and restricted range of motion. The diagnosis should be suspected when symptoms persist and characteristic MRI findings of ACL hypertrophy with preserved fiber continuity are present, and it should be confirmed arthroscopically after excluding other common intra-articular knee pathologies. When conservative treatment fails, selective arthroscopic debridement may provide effective symptom relief while preserving ACL function and knee stability.

## Figures and Tables

**Figure 1:**
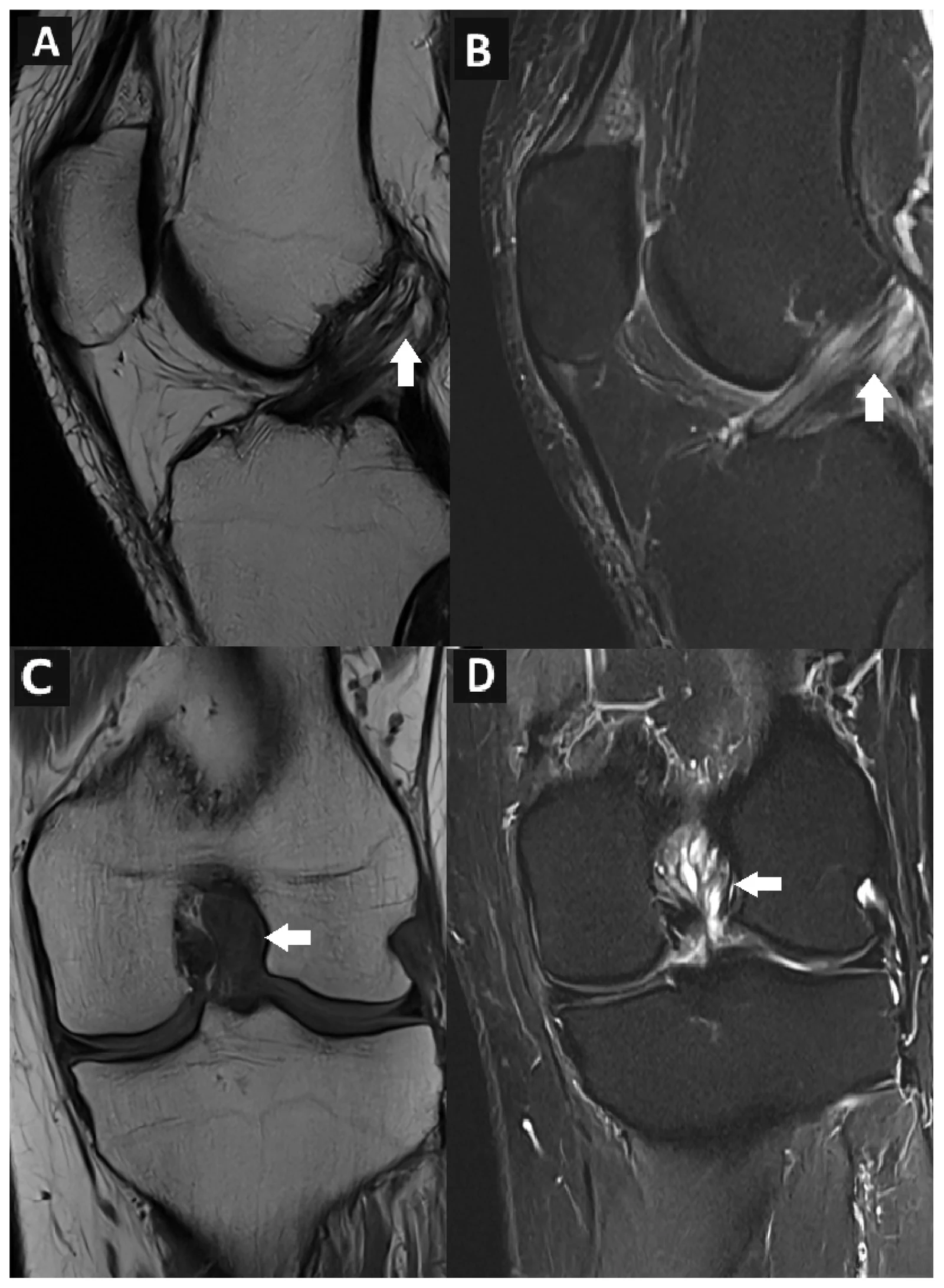
Magnetic resonance images of the knee demonstrating mucoid degeneration of the anterior cruciate ligament (ACL). Sagittal (**A**,**B**) and coronal (**C**,**D**) images reveal diffuse thickening and hypertrophy of the ACL, with increased intra-ligamentous signal intensity and preserved continuity of the ligament fibers (white arrow), findings consistent with mucoid degeneration.

**Figure 2:**
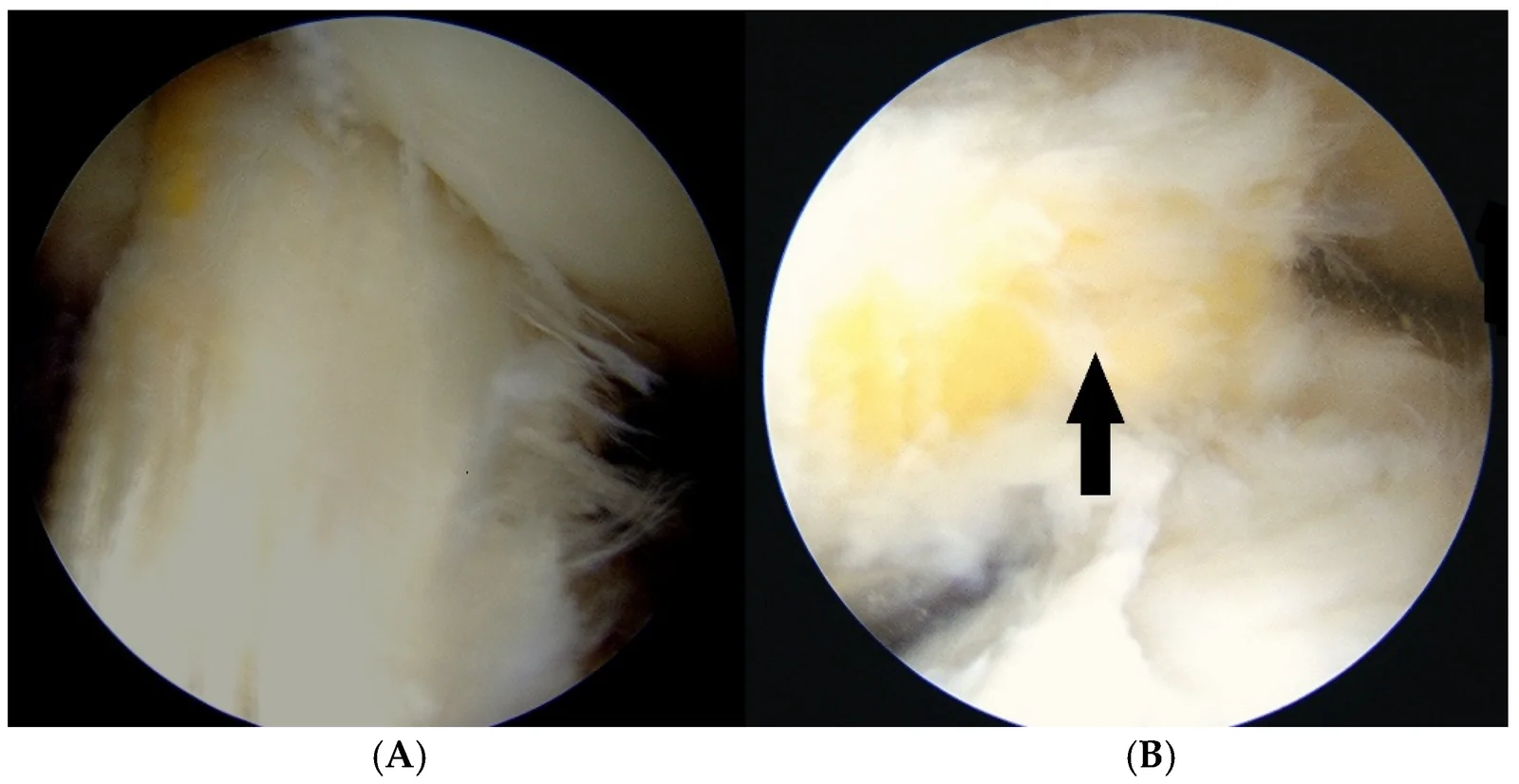
Arthroscopic views of mucoid degeneration of the anterior cruciate ligament. (**A**) Hypertrophy with loss of the normal ligament architecture causing impingement against the intercondylar notch and surrounding structures. (**B**) Selective arthroscopic debridement was performed while preserving ACL stability. Note the extensive intra-ligamentous mucoid degeneration (black arrow).

## Data Availability

The original contributions presented in this study are included in the article. Further inquiries can be directed to the corresponding author.

## Abbreviations

The following abbreviations are used in this manuscript:

ACL	Anterior cruciate ligament
MD	Mucoid degeneration
MRI	Magnetic resonance imaging
ROM	Range of motion
NSAIDs	Nonsteroidal anti-inflammatory drugs
PRP	Platelet-rich plasma
